# Supplementary material for: Hidden flaws behind expert-level accuracy of multimodal GPT-4 vision in medicine
Source: NPJ Digit Med. 2024 Jul 23;7:190. doi: 10.1038/s41746-024-01185-7 (PMC11266508; doi:10.1038/s41746-024-01185-7)
Supplement: Supplementary file 1 — Supplementary File [file 41746_2024_1185_MOESM1_ESM.pdf]

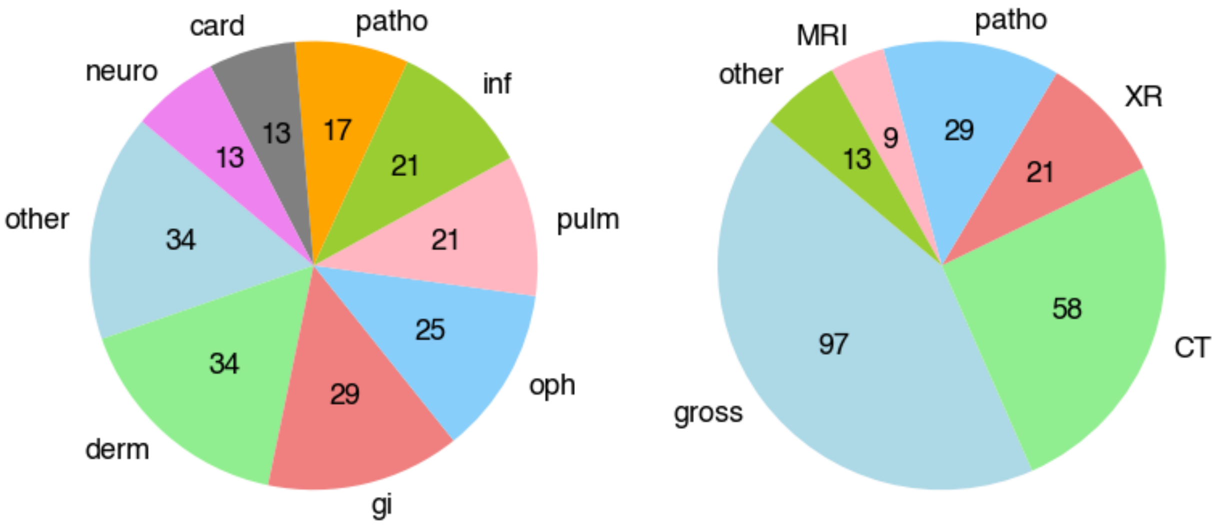


**Supplementary Figure 1**. The medical specialty and imaging modality distributions of the 207 NEJM Image Challenges used in this study. (derm: dermatology, other: other internal medicine, pulm: pulmonology, neuro: neurology, gi: gastroenterology, oph: ophthalmology, card: cardiology, inf: infectious diseases; gross: macroscopic clinical images, patho: microscopic pathology, CT: computed tomography, XR: X-ray, MRI: magnetic resonance imaging; other: other image types including electrocardiogram, echocardiogram, etc.).

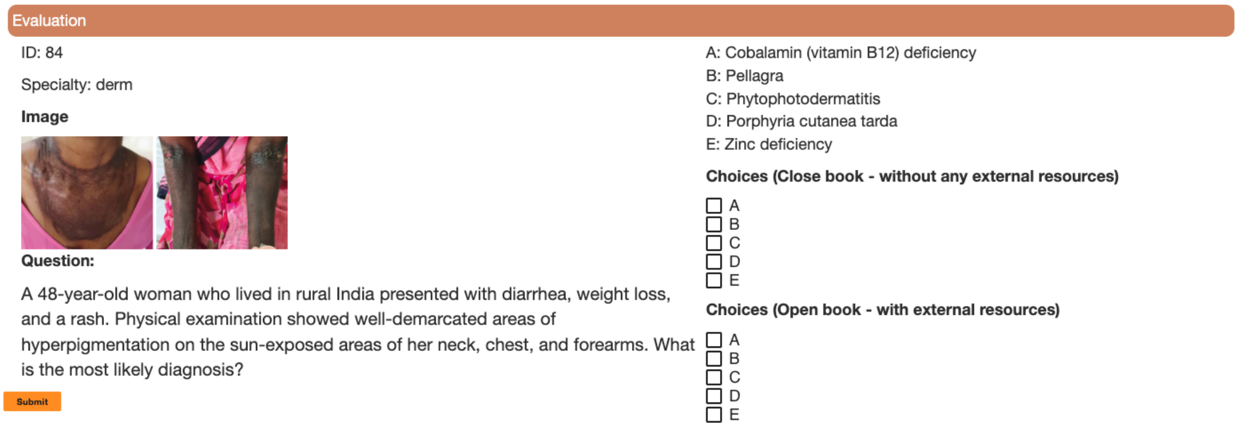


**Supplementary Figure 2**. Annotation interface for human physicians to provide their closed-book and open-book answers to NEJM Image Challenge.

**Evaluation**

ID: 19

Specialty: neuro

**Image**

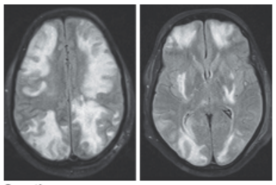

**Question:**

A 35-year-old man with IgA nephropathy presented with confusion, blurry vision, and seizures. Two weeks before presentation, he had started receiving cyclosporine. Physical examination was notable for a blood pressure of 160/80 mm Hg, drowsiness, and decreased visual acuity. A fundoscopic examinations was normal. T2-weighted magnetic resonance imaging (MRI) with fluid-attenuated inversion recovery sequencing of the head was performed. What is the most likely diagnosis?

**Choices:**

A: Acute demyelinating encephalomyelitis
B: Methanol ingestion
C: Multifocal ischemic infarcts
D: Posterior reversible encephalopathy syndrome
E: West Nile virus encephalitis

**Ground-truth explanation:**

A diagnosis of posterior reversible encephalopathy syndrome (PRES) due to cyclosporine use was made. One week after cyclosporine therapy was stopped, the patient's symptoms and MRI findings had resolved.

**Ground-truth answer:** Posterior reversible encephalopathy syndrome

Submit

**GPT4V answer:**

**Image Comprehension:** ☐ Correct ☐ Partially correct ☐ Incorrect

Reason/Comment

The images provided are T2-weighted MRI scans of a patient's brain with fluid-attenuated inversion recovery (FLAIR) sequencing. There are hyperintense (bright) regions in both images. In the left image, the hyperintensity is observed bilaterally in the occipital lobes. In the right image, the hyperintensity is also seen in the occipital lobes, with some involvement of the parietal lobes.

**Medical Knowledge Recall:** ☐ Correct ☐ Partially correct ☐ Incorrect

Reason/Comment

Acute demyelinating encephalomyelitis (ADEM): This is an inflammatory demyelinating disease of the CNS. MRI findings typically include large, poorly-defined, asymmetric white matter lesions. Methanol ingestion: Methanol poisoning can cause metabolic acidosis and visual disturbances due to its metabolites, but MRI brain findings are typically not characteristic. Multifocal ischemic infarcts: These would typically show as multiple small focal regions of restricted diffusion on diffusion-weighted MRI sequences. Posterior reversible encephalopathy syndrome (PRES): This syndrome can be associated with various conditions, including hypertension, immunosuppressive medications (like cyclosporine), and renal failure. MRI findings in PRES typically involve bilateral hyperintensities in the parietal and occipital lobes on T2/FLAIR sequences. West Nile virus encephalitis: While West Nile can cause encephalitis, the MRI findings can vary, but they do not typically present as bilateral occipital/parietal hyperintensities.

**Step-by-step Reasoning:** ☐ Correct ☐ Partially correct ☐ Incorrect

Reason/Comment

Based on the history provided, the patient has recently started taking cyclosporine. Cyclosporine is known to be associated with PRES. The MRI findings of bilateral occipital and parietal hyperintensities on T2/FLAIR sequences are characteristic of PRES. While the patient does have hypertension (a known risk factor for PRES), the other potential diagnoses either do not align with the MRI findings (like ADEM, multifocal ischemic infarcts, and West Nile virus encephalitis) or the patient's clinical history (like methanol ingestion).

**Final Answer:** ☐ True ☐ False

Answer: D. Posterior reversible encephalopathy syndrome (PRES).

**Supplementary Figure 3**. Annotation interface for human physicians to provide their judgments on whether each section (image comprehension, recall of medical knowledge, step-by-step reasoning, and the final answer) of the GPT-4V response contains errors.

**Supplementary Table 1.** The physician accuracy, GPT-4V accuracy, and the identified hidden flaws for each specialty.

| Specialty | Physician accuracy | GPT-4V accuracy | Hidden Flaws |
|---|---|---|---|
| Dermatology | 79.4% | 94.1% | 18.8% |
| Pathology | 70.6% | 82.4% | 42.9% |
| Pulmonary | 85.7% | 81.0% | 52.9% |
| Gastrointestinal | 75.9% | 79.3% | 39.1% |
| Neurology | 84.6% | 100.0% | 53.8% |
| Ophthalmology | 88.0% | 80.0% | 60.0% |
| Cardiology | 61.5% | 92.3% | 0.0% |
| Infectious diseases | 95.2% | 66.7% | 64.3% |
| Internal Medicine | 61.8% | 70.6% | 8.3% |
| All | 77.8% | 81.6% | 35.5% |

**Supplementary Table 2**. Summary of relevant studies in comparison to our evaluation.

| Evaluation | Multi-choice Accuracy | | GPT-4V Rationale Evaluation |
|---|---|---|---|
| | GPT-4V | Human Performance | |
| 92 cases from radiology and pathology websites[10] | NA | NA | Yes (all) & qualitative by ad-hoc comments |
| 133 from medical visual question answering datasets[11] | 29.9% (pathology) 50.0% (radiology) | NA | Partial (through case studies in the error analysis of GPT-4V) |
| 226 USMLE examination questions[12] | 84.2%-88.9% | NA | Partial (through case studies in the error analysis of GPT-4V) |
| 934 NEJM Image Challenge[13] | 61% (scored by GPT-4) | 49% (NEJM online anonymous respondents) | NA |
| 207 NEJM Image Challenge (this study) | 81.6% (scored by physicians) | 61.4% (medical student) 77.8% (physicians) | Yes (all 207 cases) & systematically on three distinct capabilities |

**Supplementary Data 1. NEJM Image Challenge Question 96 (12/9/21)**

A 74-year-old woman presented with a 3-day history of headaches and tongue swelling and several hours of blindness in the right eye. On examination, the tongue was discolored and ulcerated. What is the diagnosis?

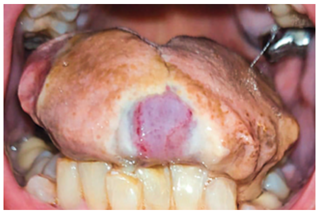

A. Carcinoma of the tongue
B. Carotid artery stenosis
C. Giant-cell arteritis
D. Oral lichen planus
E. Thromboembolic disease

**Correct Answer:** Giant-cell arteritis
**Explanation:** The correct answer is giant-cell arteritis. Lingual infarction is a rare complication of giant-cell arteritis. Her vision and tongue necrosis improved with high-dose glucocorticoids and tocilizumab.

| GPT-4V Answer | |
|---|---|
| **Image Comprhension:** The image depicts an up-close view of a person's tongue. The tongue appears swollen with discoloration that is unevenly distributed. Notably, there is a prominent ulcerated region that is reddish-purple in color, surrounded by areas of paler discoloration. This particular ulceration stands out due to its size and distinct coloration. Additionally, there's a dental instrument present, suggesting the image was taken during a clinical examination. | **Annotation:** Correct<br>**Comment:** All correct |
| **Recall of Medical Knowledge:** Carcinoma of the tongue: This refers to a malignant tumor of the tongue. Signs include persistent tongue pain, a lump or sore on the tongue that doesn't heal, and difficulty chewing or swallowing. Ulceration can be a feature of tongue cancer. Carotid artery stenosis: This involves narrowing of the carotid arteries, the primary arteries supplying blood to the brain. Symptoms might include sudden numbness or weakness, difficulty speaking, vision problems, and dizziness. However, it does not typically cause tongue ulceration. Giant-cell arteritis (GCA): An inflammatory condition affecting the lining of your arteries, especially the arteries in the temples. Symptoms include headaches, jaw pain, vision problems, and sometimes tongue pain. If left untreated, GCA can lead to blindness. Oral lichen planus: This is an inflammatory condition that can affect the mucous membranes inside the mouth. Oral lichen planus can cause white patches, redness, and ulcers. Thromboembolic disease: This refers to a condition where blood clots form in the bloodstream and block blood flow. Depending on the location, it could lead to various symptoms. While it might explain the blindness if a clot were to affect the blood supply to the eye, it doesn't directly correlate with tongue symptoms. | **Annotation:** Correct<br>**Comment:** All correct |
| **Step-by-step Reasoning:** Given the patient's presentation with a 3-day history of headaches, tongue swelling, and several hours of blindness in the right eye, and considering the image of the ulcerated and discolored tongue, we can proceed with the following reasoning:<br>The ulceration on the tongue doesn't immediately point to carotid artery stenosis or thromboembolic disease as the primary cause. Carcinoma of the tongue could account for the ulceration, but it doesn't explain the sudden blindness or the headaches. Oral lichen planus could potentially account for tongue symptoms but wouldn't necessarily explain the blindness. Giant-cell arteritis (GCA), on the other hand, can cause headaches, vision problems (including blindness), and tongue pain or discoloration due to inflammation of the arteries. In severe cases, the reduced blood flow could cause tongue ulceration. | **Annotation:** Correct<br>**Comment:** All correct |
| **Final Answer:** Given the combination of symptoms and the image, the most fitting diagnosis would be: C. Giant-cell arteritis. | **Annotation:** Correct |

**Supplementary Data 2. NEJM Image Challenge Question 12 (7/20/23)**

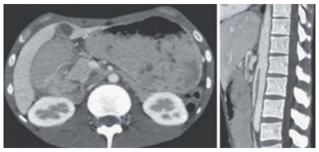

A 26-year-old man from Somalia presented with a 5-month history of dry cough, night sweats, and unintentional weight loss of 18 kg. During this period, epigastric pain and postprandial vomiting had also developed. His BMI was 11. On examination, he was cachectic with abdominal distention and diffuse tenderness to palpation. On the basis of chest imaging and sputum studies, a diagnosis of pulmonary tuberculosis was made, and intravenous antituberculous treatment was initiated. However, he continued to have postprandial vomiting. Contrast-enhanced CT of the abdomen was obtained. What is the cause of his abdominal symptoms?

A. Chronic mesenteric ischemia
B. Gastric tuberculosis
C. Gastrointestinal lymphoma
D. Intussusception
E. Superior mesenteric artery syndrome

**Correct Answer:** Superior mesenteric artery syndrome
**Explanation:** The correct answer is superior mesenteric artery syndrome. The CT scan of the abdomen showed a paucity of mesenteric and subcutaneous fat, dilatation of the stomach, and compression of the duodenum between the superior mesenteric artery and the aorta, with an aortomesenteric distance of 3 mm (reference range, 10 to 20) and an aortomesenteric angle of 7° (reference range, 45 to 60). A diagnosis of superior mesenteric artery syndrome due to cachexia from tuberculosis was made.

**GPT-4V Answer**

| | |
|---|---|
| **Image Comprhension:** The provided image consists of three separate sections of a contrast-enhanced CT scan, primarily focusing on the abdomen. The scans reveal:<br>The first section (leftmost): A transverse view of the upper abdomen which shows a dilated stomach with retained content and significant distension. The surrounding vessels and structures appear normal.<br>The second section (middle): Another transverse view slightly below the first section, showing continued stomach dilation and distension. The duodenum appears to be compressed.<br>The third section (rightmost): A sagittal section of the abdomen, which provides a clearer view of the compressed duodenum between the aorta and the superior mesenteric artery. | **Annotation:** Partially correct<br>**Comment:** There are only 2 CT images, thus the GPT is misinterpreting this fact. |
| **Recall of Medical Knowledge:** Superior Mesenteric Artery (SMA) syndrome is a rare gastrointestinal condition where the third part of the duodenum is compressed between the aorta and the superior mesenteric artery. This compression results in partial or complete duodenal obstruction. The syndrome is often associated with conditions leading to rapid weight loss, which causes a loss of the mesenteric fat pad, thereby reducing the angle between the SMA and the aorta and leading to compression of the third part of the duodenum. Symptoms include postprandial vomiting, abdominal pain, and weight loss. | **Annotation:** Correct |
| **Step-by-step Reasoning:** The patient's history of rapid weight loss (18 kg) and the resultant low BMI of 11 supports the notion of a diminished mesenteric fat pad. The CT images show a dilated stomach with retained contents, which suggests a form of gastric outflow obstruction. The sagittal section of the abdomen distinctly reveals the third part of the duodenum being compressed between the aorta and the superior mesenteric artery. This anatomical change, combined with the patient's symptoms and clinical presentation, leads us to the diagnosis. | **Annotation:** Correct |
| **Final Answer:** Answer: E. Superior mesenteric artery syndrome. | **Annotation:** Correct |

**Supplementary Data 3. NEJM Image Challenge Question 21 (5/18/23)**

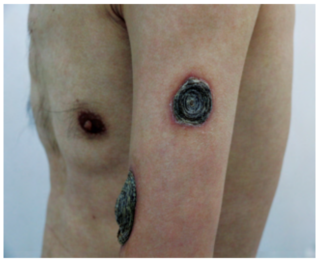

A 44-year-old man with a history of human immunodeficiency virus infection and intermittent adherence to antiretroviral therapy presented to the infectious disease clinic with a 1-month history of fevers and pruritic skin lesions. Physical examination was notable for blackish-brown lamellated plaques on the limbs and scalp. Laboratory studies were notable for a CD4 cell count of 86 per cubic millimeter (reference range, 414 to 1123) and a rapid plasmin reagin titer of 1:32. A skin biopsy of the left forearm showed diffuse dermal lymphocytes and histiocytes admixed with a plasma-cell infiltrate. What is the most likely diagnosis?

A. Erythema annulare centrifugum
B. Erythema gyratum repens
C. Erythema migrans (Lyme disease)
D. Lupus vulgaris (cutaneous tuberculosis)
E. Malignant syphilis

**Correct Answer:** Malignant syphilis
**Explanation:** The correct answer is malignant syphilis. A Treponema pallidum particle agglutination assay was also positive. Malignant syphilis is an uncommon form of secondary syphilis that primarily affects immunocompromised persons. The skin manifestations are varied, ranging from ulceronodular lesions to oyster shell-like crusts, as seen in this patient. After a 2-week course of intravenous antimicrobial therapy, the skin lesions and fevers abated.

| GPT-4V Answer | |
|---|---|
| **Image Comprhension:** The image displays a close-up of skin, revealing two main features: A raised blackish-brown plaque with a lamellated appearance. This lesion appears to have concentric rings, giving it a layered look. There is also a darker, brownish circular mark on the skin, which may or may not be related to the patient's presenting symptoms. | **Annotation:** Incorrect<br>**Comment:** The more distal plaque likely represents the same finding as the proximal lesion. |
| **Recall of Medical Knowledge:** Erythema annulare centrifugum: Presents as annular (ring-shaped) red plaques that tend to be itchy. Erythema gyratum repens: Characterized by concentric rings of erythema that give a wood-grain appearance. It can be associated with internal malignancies. Erythema migrans (Lyme disease): Presents as a red, expanding rash with a clear center, resembling a "bull's-eye." Lupus vulgaris (cutaneous tuberculosis): Chronic and progressive form of skin TB, with reddish-brown nodules and plaques. Malignant syphilis: Also known as lues maligna, is a rare form of secondary syphilis. It manifests as papules, nodules, or ulcers, often covered by a grayish-white membrane or necrotic tissue. Given the patient's HIV status and the described lab findings, this condition becomes more pertinent. | **Annotation:** Correct |
| **Step-by-step Reasoning:** Given the description and the image, the skin lesion does not resemble Erythema annulare centrifugum, Erythema gyratum repens, or Erythema migrans. While Lupus vulgaris could present with nodules and plaques, the blackish-brown lamellated appearance of the lesion is not typical for cutaneous tuberculosis. Malignant syphilis, on the other hand, can have varied manifestations, including the described lesion appearance. Moreover, the patient's HIV infection and low CD4 cell count make him more susceptible to atypical infections, including unusual presentations of syphilis. The rapid plasmin reagin (RPR) titer of 1:32 further supports a syphilis diagnosis. | **Annotation:** Correct |
| **Final Answer:** Based on the image comprehension, recalled medical knowledge, and step-by-step reasoning, the most likely diagnosis for this patient is E.Malignant syphilis. | **Annotation:** Correct |

**Supplementary Data 4. NEJM Image Challenge Question 95 (12/16/21)**

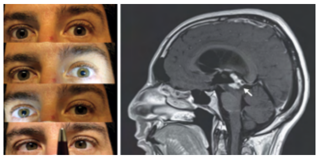

A 37-year-old man presented to the neurosurgery clinic with blurry vision, headache, and difficulty looking upward. On exam, he had diplopia, upward gaze palsy, and vertical misalignment of the eyes. His pupils only minimally constricted to light but constricted to near objects. What is the name of this pupillary exam finding?

A. Adie's pupil
B. Anisocoria
C. Argyll Robertson pupil
D. Light-near dissociation
E. Relative afferent pupillary defect (e.g., Marcus Gunn pupil)

**Correct Answer:** Light-near dissociation
**Explanation:** Magnetic resonance imaging of the patient's brain showed an enhancing dorsal midbrain lesion; biopsy later confirmed nonsecreting germinoma. Pupillary light-near dissociation is characterized by the absence of constriction to light with an intact accommodation reflex. Causes of light-near dissociation include dorsal rostral midbrain lesions, as in this case, and neurosyphilis, though the Argyll Robertson pupils associated with syphilis are also typically miotic and irregular at baseline.

---

**GPT-4V Answer**

**Image Comprhension:** The image consists of two parts. On the left side, there are multiple close-up photographs of a person's eyes. In these photographs, the pupils show variations in constriction and dilation. The pupils appear to be different in size in some images, and in others, the eye appears to have difficulty looking in certain directions, particularly upwards. The images depict the clinical findings mentioned: blurry vision, difficulty in upward gaze, and possibly the pupillary response to light and near objects.
On the right side, there is an MRI scan of what appears to be a sagittal section of a head. There is an arrow pointing to a specific region within the brain, suggesting some kind of lesion or abnormality in that area.

**Annotation:** Incorrect
**Comment:** The key to the image on the left is the lack of constriction to light but constriction to near objects. GPT referred to the vignette, but failed to mention where the text explicitly described the findings: "His pupils only minimally constricted to light but constricted to near objects." The interpretation of the left images incorrectly claim to depict: 1. Blurry vision - this is a subjective complaint and cannot be demonstrated on image unless it shows us a blurry image. 2. Difficulty with upward gaze - None of the images demonstrate that the subject is attempting upgaze; 3. GPT states POSSIBLY the pupillary response to light and near objects - This does not describe a finding at all, whether it is abnormal or not. Regarding the right image (the saggital view of the brain MRI, iit is correct that is it a sagittal section of the head, although we typically say MRI's are of the brain. It is correct there is an arrow pointing to a specific region of the brain, but it does not state what the location is. And it says the arrow suggests there is some kind of lesion or abnormality in that (undefined) region. There is, in fact, an enhancing lesion in the dorsal midbrain.

| | |
|---|---|
| **Recall of Medical Knowledge:** Adie's pupil: A neurological disorder characterized by a tonically dilated pupil that reacts slowly to light but shows a more normal response to accommodation (i.e., looking at near objects). Anisocoria: A condition characterized by an unequal size of the eye's pupils. Argyll Robertson pupil: Pupils that are small, irregular in shape, and bilaterally constricted. They do not react to direct light but constrict with accommodation. Light-near dissociation: The pupils do not react or react minimally to direct light but constrict when the eye focuses on a near object. This is seen in conditions such as neurosyphilis and third nerve palsy, among others. Relative afferent pupillary defect (Marcus Gunn pupil): A condition in which the direct light reflex is decreased or absent in one eye when a light is shone into it, while the consensual reflex (response of the other eye) is normal. | **Annotation:** Partially correct<br>**Comment:** Its description of Relative afferent pupillary defect is not correct. It is close, but it should be that when light is shown in the affected eye, there is no or slight constriction of both pupils. When shown in the unaffected eye, there is normal constriction of both pupils. So when performing the swinging lamp test, the pupils will appear to dilate when swinging the light to the affected side and constrict when swinging the light to the unaffected side. |
| **Step-by-step Reasoning:** From the image and description, the person's pupils only minimally constrict to light but constrict to near objects. This pattern of response is consistent with light-near dissociation. Given the other symptoms, including difficulty looking upward and vertical misalignment, it suggests a pathology that could be associated with third nerve palsy or other neurological issues. The MRI might show a lesion or abnormality causing these symptoms, but the specific clinical findings regarding the pupils align with light-near dissociation. | **Annotation:** Partially correct<br>**Comment:** It correctly interpreted the pupillary findings which was explicitly described in the text. Yes, this represents light near dissociation. The trick with this question is that Argyll Robertson Pupil is also a form of light near dissociation and one that many clinicians associate with the clinical findings. However, it is specific to an etiology of neurosyphilis. However this imaging finding showing a dorsal midbrain enhancing lesion can also cause the same pupilary findings (light near dissociation) as Argyll Robertson pupil, but when associated with a lesion due to something other than neurosyphilis, which in this case we can assum is due to a mass lesion, it is referred to as the more broad light-near dissociation. GPT was still uncertain about the presence of a lesion causing these symptoms. |
| **Final Answer:** Answer: D. Light-near dissociation. | **Annotation:** Correct |
